# All‐cause mortality and Japan's early countermeasures: Authors' reply

**DOI:** 10.1002/hsr2.2244

**Published:** 2024-07-04

**Authors:** Yudai Kaneda, Kenzo Takahashi

**Affiliations:** ^1^ School of Medicine Hokkaido University Hokkaido Japan; ^2^ Teikyo University Graduate School of Public Health Tokyo Japan

We appreciate the opportunity to respond to the points raised by Søren Roest Korsgaard regarding our paper on the assessment of Japan's early response to the COVID‐19 pandemic.^1^ The letter notably emphasizes Japan's mortality outcomes, suggesting a comparative success against global standards based on the direct fatalities from COVID‐19.^1^ This perspective, while valuable, may overlook the broader spectrum of impacts that cannot be explained without considering aspects such as excess mortality, engendered by the COVID‐19 pandemic and the response measures implemented.

The argument posited by Korsgaard, focusing primarily on COVID‐19 as a direct cause of death,^1^ merits expansion to encompass the nuanced effects of the pandemic response on the broader population. According to the Ministry of Health, Labor and Welfare's vital statistics, the death rate per 100,000 population in 2022 increased by 166 compared to 2019, with notable rises in deaths due to senility (an increase of 48, or 49%), heart diseases (an increase of 22, or 14%), and aspiration pneumonia (an increase of 13, or 41%) closely linked with senility, whereas the increase in COVID‐19 related deaths was only 38.^2^ This represents a 3.4‐fold increase in deaths from causes other than COVID‐19,^2^ underscoring that while Korsgaard's observations are valid when considering COVID‐19 as a direct cause of death,^1^ a comprehensive evaluation must also take into account of the indirect impacts of the response measures.

Additionally, Korsgaard's assertion that Japan did not implement a lockdown is not entirely accurate.^1^ In reality, Japan was subject to varying restrictions, starting with a state of emergency declared on April 7, 2020, for seven prefectures: Tokyo, Kanagawa, Saitama, Chiba, Osaka, Hyogo, and Fukuoka.^3^ This declaration was expanded nationwide on April 16, 2020, and the state of emergency was declared four times, specifically targeting regions where the COVID‐19 outbreak was severe, totaling 275 days (periods: 2020/4/7 to 2020/5/25, 2021/1/8 to 2021/3/21, 2021/4/25 to 2021/6/20, and 2021/7/12 to 2021/9/30).^3^ Semi‐emergency coronavirus measures were also in effect from 2021/4/5 to 2021/9/30 and from 2022/1/9 to 2022/3/21.^3^ During the periods, people in Japan were requested to refrain from unnecessary and nonurgent gatherings and outings,^4^, ^5^ and there were reports of elective surgical procedures being canceled and a reluctance to seek medical consultations,^6^, ^7^ which can be regarded as a voluntary‐based lockdown.^8^ The increased proportions of deaths due to senility, heart disease, and aspiration pneumonia, conditions that are more likely to occur in elderly individuals with decreased physical strength,^2^ cannot be overlooked in light of the impact of the state of emergency declarations. This extensive duration under restrictions underscores the critical need to assess the indirect health impacts of pandemic measures, alongside the direct outcomes of COVID‐19 fatalities.

Thus, it is premature to conclusively declare Japan's COVID‐19 measures a success based solely on hard outcomes; our discussion instead centers on the inadequacy of the scientific approach employed in the process, which we believe is essential to review for the preparedness to the coming pandemic. For instance, despite conclusions from reviews on novel influenza suggesting that uniform school closures were unnecessary,^9^ the decision by the Prime Minister to enforce nationwide school closures in the context of COVID‐19 inadvertently hindered healthcare workers' ability to attend their duties, resulting in poor outcome evaluation “the potential negative consequences for children and parents.”^10^, ^11^ Furthermore, while the letter might have an impression that we disapprove of the vaccine rollout,^1^ it is crucial to acknowledge that Japan has indeed succeeded in the rapid deployment of vaccines, largely thanks to the significant contributions of local governments.^12^ Evaluating these processes and retaining lessons from them is of paramount importance.

In conclusion, our objective extends beyond a simplistic binary assessment of Japan's pandemic response. We aim to underscore the importance of a nuanced, process‐oriented analysis that can provide actionable insights for future pandemic preparedness. Through a comprehensive examination of decision‐making, policy implementation, and adaptability to evolving scientific evidence, we advocate for an approach that enhances our collective resilience against future public health challenges.

## AUTHOR CONTRIBUTIONS

Conception and designing of the study, data collecting, and writing this paper; Yudai Kaneda. Critical revision of the paper; Kenzo Takahashi. All the authors read the final draft and approved submission.

## CONFLICT OF INTEREST STATEMENT

The authors declare no conflict of interest.

## TRANSPARENCY STATEMENT

The lead author (Yudai Kaneda) affirms that this manuscript is an honest, accurate, and transparent account of the study being reported; that no important aspects of the study have been omitted; and that any discrepancies from the study as planned (and, if relevant, registered) have been explained.

## Data Availability

Data sharing is not applicable to this article as no new data were created or analyzed in this study.
